# Improving Global Healthcare and Reducing Costs Using Second-Generation Artificial Intelligence-Based Digital Pills: A Market Disruptor

**DOI:** 10.3390/ijerph18020811

**Published:** 2021-01-19

**Authors:** Yaron Ilan

**Affiliations:** Department of Medicine, The Hebrew University of Jerusalem-Hadassah Medical Center, Jerusalem 12000, Israel; ilan@hadassah.org.il

**Keywords:** digital health, healthcare costs, generic drugs, chronic disease, compensatory response

## Abstract

*Background and Aims:* Improving global health requires making current and future drugs more effective and affordable. While healthcare systems around the world are faced with increasing costs, branded and generic drug companies are facing the challenge of creating market differentiators. Two of the problems associated with the partial or complete loss of response to chronic medications are a lack of adherence and compensatory responses to chronic drug administration, which leads to tolerance and loss of effectiveness. *Approach and Results:* First-generation artificial intelligence (AI) systems do not address these needs and suffer from a low adoption rate by patients and clinicians. Second-generation AI systems are focused on a single subject and on improving patients’ clinical outcomes. The digital pill, which combines a personalized second-generation AI system with a branded or generic drug, improves the patient response to drugs by increasing adherence and overcoming the loss of response to chronic medications. By improving the effectiveness of drugs, the digital pill reduces healthcare costs and increases end-user adoption. The digital pill also provides a market differentiator for branded and generic drug companies. *Conclusions:* Implementing the use of a digital pill is expected to reduce healthcare costs, providing advantages for all the players in the healthcare system including patients, clinicians, healthcare authorities, insurance companies, and drug manufacturers. The described business model for the digital pill is based on distributing the savings across all stakeholders, thereby enabling improved global health.

## 1. Introduction

Healthcare systems are moving towards a population health approach. The cost of healthcare for patients with chronic diseases is a major burden on these systems [1]. Patients with non-communicable diseases (NCDs) in the United States are estimated to face annual expenses of above USD 3 trillion [2]. Approximately 70% of deaths and 40% of costs are attributable to NCDs [3], and while most chronic diseases have potentially effective solutions, the partial or complete loss of response to chronic medications leads to increased morbidity from these diseases [4]. 

There is a growing need to decrease costs and improve quality while treating patients with common chronic disorders [1]. At least half of the world’s population cannot obtain essential health services, and from a population health perspective, the lack of accessibility of a large proportion of the population to chronic drugs is a major hurdle to improving overall health [5]. Health authorities and insurance companies both share these burdens, which impact personal and societal health. Although the lack of resources could be partially answered by using biosimilars and generics [6], the overcrowded market of generics continues to be a major challenge for companies. Similarly, the overall response rate to innovative medications for NCDs is far from ideal, leaving high percentages of non-responsive patients and creating an economic burden for healthcare systems. 

Although digital health systems have been used over the last decade, defined as first-generation artificial intelligence (AI), their implementation in everyday care is limited. This aim of the present study is to review some of the health and economic challenges faced by healthcare systems and drug companies, both innovative and generic, and to discuss the use of digital pills, which are drugs regulated by second-generation artificial intelligence (AI) systems, as a means to deal with some of these challenges. The use of digital pills is intended to reduce healthcare costs, improve the overall health of the population, and increase revenues for drug manufacturers.

## 2. Challenges of Healthcare Systems in Dealing with Chronic Disease: High Costs and Lack of Effective and Affordable Therapies 

Chronic NCDs are a significant cause of morbidity and mortality worldwide [3]. Cardiovascular diseases account for most NCD deaths, followed by cancers, respiratory diseases, and diabetes [7]. These four groups of diseases account for over 80% of all premature NCD deaths [8]. Mental health conditions and cardiovascular diseases impose the highest burdens, followed by cancer, diabetes, and chronic respiratory diseases [2].

NCDs have major economic impacts worldwide. Modeling NCDs according to a human capital-augmented production function was found to impact the effects of projected disease prevalence. Chronic disease generally develops long before a fatal outcome, leading to multiple years of poor health and a high cost burden [9]. Deaths from NCDs in working-age individuals are associated with a direct loss, as physical capital only partially substitutes for the loss of human capital in the production process. Working-age individuals who suffer from NCDs but do not die because of their disease tend to be less productive. NCD interventions, such as medical treatments and prevention, are associated with high costs. A total loss of USD 94 trillion was projected for all NCDs for the next four decades [2]. In sub-Saharan Africa, the economic burden of NCDs is high and associated with catastrophic spending, which further aggravates poverty [7].

## 3. Loss of Effectiveness of Chronic Drugs: Low Adherence and the Development of Drug-Resistance 

Two major problems associated with the partial or complete loss of response to chronic medications are the lack of adherence and compensatory responses to the triggers imposed on biological systems by the chronic administrations of drugs [4]. 

Low adherence is a common problem for many NCDs. In the real world, low statin adherence during the first year after an acute myocardial infarction is associated with increased mortality during the second year [10]. Less than half of severely asthmatic patients adhere to inhaled treatments [11], while over 40% of hypertensive patients show non-adherence [12].

The partial or complete loss of response to chronic medications involves multiple different mechanisms for different drugs. Over one-third of epileptics develop resistance to anti-epileptic drugs [13]. A similar percentage of patients with depression develop resistance to anti-depressants [14]. Diuretic resistance and insulin resistance are common among patients with congestive heart failure and diabetes, respectively [15,16]. Cancer drug resistance is a major obstacle for the treatment of multiple malignancies [17,18]. One-third of patients with rheumatoid arthritis (RA) display an inadequate primary response to anti-tumor necrosis factor (TNF)-based drugs [19]. A primary loss of response to anti-TNFs also occurs in 40% of patients with inflammatory bowel disease [20]. A secondary loss of response, following an initial effect, occurs in 25–61% of these patients [21,22,23]. 

Inter- and intra-patient variability in the response to chronic drugs further impacts the clinical response to chronically administered therapies [24]. Diversity in drug responses and drug toxicity are associated with ethnic variances, which manifest as alterations in the pharmacokinetics and pharmacodynamics of drugs, as well as with the pharmacogenomics of the host [25]. Intra- and inter-patient variability in the response to drugs and the heterogeneity of disease, along with the marked diversity of the compensatory mechanisms, all contribute to drug resistance in a time-dependent way [4,24,26]. Loss of effectiveness is also associated with increased toxicity, which may result in attempts to increase dosages, and is a major cause of the low adherence of patients to chronic medications.

## 4. The Challenge Faced by Branded and Generic Drug Companies: Difficulties in Setting Product Differentiators

The majority of chronic diseases are caused by risk factors, which are mostly preventable. Effective interventions to reduce and treat these risks are available for most NCDs [9]. Two obstacles to implementing large population treatment strategies are the need for affordable generics and the development of drug resistance towards both generics and innovative agents [4,27,28]. 

Each year, millions of patients worldwide are unable to afford essential life-saving medicines. The cost of prescription drugs, including life-saving drugs, is rising far faster than inflation [29]. Switching from branded to generic medications is a common cost-containment measure for healthcare systems [30]. Increased incentives for generic drug use are proposed as a means for improving overall health [31,32]. 

Analyses of the clinical and economic consequences of switching from branded to generic medications have focused on patient attitudes and adherence, clinical and safety outcomes, and cost and resource utilization. Several studies have suggested that switching negatively influences adherence. Other studies have suggested that generic switching is associated with poorer clinical outcomes and more adverse events [30]. Immune-mediated inflammatory diseases (IMIDs) are chronic conditions associated with a marked disease burden. Biologic medicines have contributed significantly to improving the clinical outcomes of IMIDs. However, their high cost has limited their accessibility and delayed their initial use. Using cost-efficient biosimilar anti-TNFs over the last few years has enabled more patients to access these therapies earlier and for longer, potentially reducing the long-term disease burden [33].

It is estimated that between 2014 and 2020, USD 259 billion in worldwide pharmaceutical sales were at risk due to patent expiration. Loss of exclusivity (LOE) could mean the end of a product’s value and revenue stream. LOE almost always causes a precipitous decline in sales for small-molecule drugs, and brand unit share dips by 16%, on average, by the time generics have been on the market for one year [34]. The strategies taken to overcome losses include preserving brand equity and patient loyalty, creating an over-the-counter formulation, or launching generic drugs themselves. These strategies allow branded companies to retain a higher portion of a product’s value with relatively low implementation costs. 

However, generic companies are faced with high competition and an inability to create clear market differentiators for their products. Margins for generics are continuously being reduced due to the competitive nature of this market. Pricing pressures increase as the number of generic competitors rises; generic-to-brand price ratios tend to remain above 50% when only one or two generics are available, but they dip below 25% with five or more generics [34]. The Competition and Markets Authority (CMA) in the United Kingdom has accused generic drug companies of entering into an anti-competitive “pay for delay” agreement, which may have generated extra costs for the national health systems [35]. While the use of generics reduces costs for healthcare systems, it does not resolve the issue of drug resistance. The development of a partial or complete lack of responsiveness is common for both branded drugs and generics.

## 5. First-Generation AI Systems: Lack of Effect on Patient Outcomes and Low Adoption by Patients and Clinicians

First-generation AI systems largely focus on clinical decision-making through big data analysis and developing algorithms for diagnosis and treatment [36]. While these systems were shown to be beneficial in some cases, they commonly failed to translate the algorithms into an improved clinical response, leading to a low adoption rate by patients. AI trained by big data may recognize patterns that are difficult for humans to deduce by observation [37]. Concerns regarding unacceptable results, problems of data appropriateness, and the risk of biases while counting inappropriate confounding variables are some of the challenges of these systems [38]. First-generation systems commonly do not provide understandable decision-making algorithms [38]. The trade-off between performance and explicability implies that the best-performing models are often the least explicable, whereas linear regression or decision tree models, which show poorer performance, are more explicable [38,39,40,41,42]. These factors further reduce their adoption by clinicians.

The lack of a clear effect on outcomes is a major reason for the non-adoption of these systems by both patients and caregivers. The AI “quadruple aim” of improving care, improving population health, reducing healthcare costs, and improving the work life of clinicians is not achieved by the majority of first-generation systems [43]. The improved accuracy in data analysis sought by most first-generation systems does not necessarily represent better clinical efficacy [44]. 

## 6. Second-Generation AI Systems: Focus on Improving Clinical Outcomes for a Single Subject 

Second-generation AI systems are being developed with the aim of overcoming several of the hurdles faced by first-generation platforms. Second-generation systems focus on a single subject and on improving patients’ clinical outcomes [42]. The personalized closed-loop system used by these systems is designed to improve end-organ function, overcome tolerance and loss of effectiveness, and improve the patient’s response to chronic drugs. By focusing on the patient’s clinical outcome, second-generation systems ensure improved adherence to chronic drugs and a sustainable response to chronic medications, while overcoming compensatory mechanisms associated with disease progression and drug resistance [4]. They are expected to improve the adoption of AI systems by both patients and clinicians [42].

First-generation systems were intended to endorse the 4P model of medicine: predictive, preventive, personalized, and participatory, providing patient autonomy [45]. Second-generation AI systems are designed to add a “5th P” progress, the improvement of a clinical outcome in a subject-tailored manner. Rather than analyzing data to support diagnoses, predictions, or tailoring therapies, second-generation systems focus on improving a clinically meaningful outcome [42]. 

Patient engagement, wherein patients take ownership for their health, can be assisted by these systems [1]. However, using mobile phones as reminders for medication is insufficient for improving adherence. An analysis of studies using mobile phones with patients receiving anti-retroviral therapy showed that only 41% of the studies demonstrated a positive effect for adherence, with only 12% indicating improved retention [46].

Second-generation systems are aimed at improving outcomes while reducing side effects, which are the two most important parameters for patients and their caregivers. To overcome the hurdle of biases induced by big data, these systems implement an *n* = 1 concept in a personalized therapeutic regimen. The focus of the algorithm is to improve the clinically meaningful outcome for an individual subject [42].

A second-generation system was recently described as being based on the introduction of individualized variability signatures into an algorithm for improving the beneficial effects of chronic therapeutic interventions [42]. The system is designed to overcome the compensatory mechanisms associated with drug resistance and disease progression, to ensure sustainable beneficial effects from medications. 

Regular fixed regimens for the administration of chronic drugs may not be compatible with physiological variabilities in biology, and this can underlie the loss of response to chronic drugs [26,28,47,48,49]. However, introducing variability into therapeutic regimens can improve the response to drugs [26,27,28,49,50,51,52,53,54,55,56,57,58,59]. Dose escalations, reductions, and intermittent dosing with drug holidays exert clinical benefits while minimizing adverse effects [20,60,61,62,63]. A prospective trial of patients with inflammatory bowel disease treated with anti-TNFs showed a loss of clinical response in 36% of patients on fixed dosing, compared with only 13% in patients on variable dosing regimens [64]. Real-world data support the beneficial effects of drug holidays and dose escalation/reduction [24,28,29,49,50,54,55,56,57,65,66]. Introducing variability into biological systems may improve their overall function and correct dysfunctions that underlie diseases [26,67]. Both the type and magnitude of variability in a biological system can be personalized by continuously quantifying individualized variability patterns and implementing them into algorithms [4,27,28,49,50,51,52,54,68]. 

A stepwise approach was used to establish second-generation AI systems [27,42,59,68,69]. In Version 1.0, the effect of introducing variability into the therapeutic regimens of patients who stopped responding to chronic drugs was explored using pseudo-random number generators that introduced variability in times of administration and dosages within an approved range [70]. Ongoing clinical trials (NCT03843697; NCT03747705) are evaluating these regimens in patients with inflammatory bowel disease who have lost their response to anti-TNFs, and those with drug-resistant epilepsy. 

Version 2.0 comprises a closed-loop algorithm that receives inputs based on clinical outcomes [42,69]. Version 3.0 implements host- and disease-related patterns of variability that are quantified in a personalized manner and implemented into a true-random number generator. Genotypic and phenotypic parameters are deliberately ignored by the system, as the total sums of the effects of all potential factors on the selected outcome are being considered without splitting them into individual variables. This contrasts with most first-generation AIs, which dichotomize and quantify only some of these parameters. The second-generation system adapts itself to a sum of all parameters via their effects on the clinical outcome in a single subject. Continuously individualized dynamic and unpredictable changes in disease, host response, and environmental triggers are all accounted for through their effects on the selected endpoints [42].

## 7. Reducing Healthcare Costs through Second-Generation AI: Using the Digital Pill to Improve Adherence and Overcome Loss of Response to Chronic Drugs

The digital pill comprises a branded or generic drug with a second-generation AI system that overcomes the partial or complete loss of response and adherence to the effect of chronic medications in a personalized way. It includes a user-friendly app that the patient can download to a cell phone to receive an easy-to-follow therapeutic regimen.

The reduction in healthcare costs using the digital pill is achieved by improving the response to existing medications. The digital pill supports the use of generics and biosimilars for NCDs by improving their clinical effectiveness. The digital pill overcomes the primary and secondary loss of response and improves adherence to chronic drugs [26,27,42,49,50,51,54,58]. The increase in response rate to chronic medications reduces expenses by saving on the unnecessary admission of patients who are no longer responding to their chronic medications, and the need to introduce patients to newer and, in most cases, more expensive agents.

Improving overall adherence and response to chronic medications is expected to reduce the economic burden for patients, payers, and health authorities. It leads to improved global health, for example, by improving response and patient adherence to anti-diabetics, anti-hypertensive, and secondary prevention drugs in patients with cardiovascular diseases. Better responses also reduce the loss of working days, further contributing to the global economy [71,72,73]. By focusing on clinical outcomes and the reduction of side effects, second-generation AI systems are likely to increase their adoption by patients and clinicians, supporting prolonged adherence to and sustainable effects from chronic drugs.

**Figure 1:**
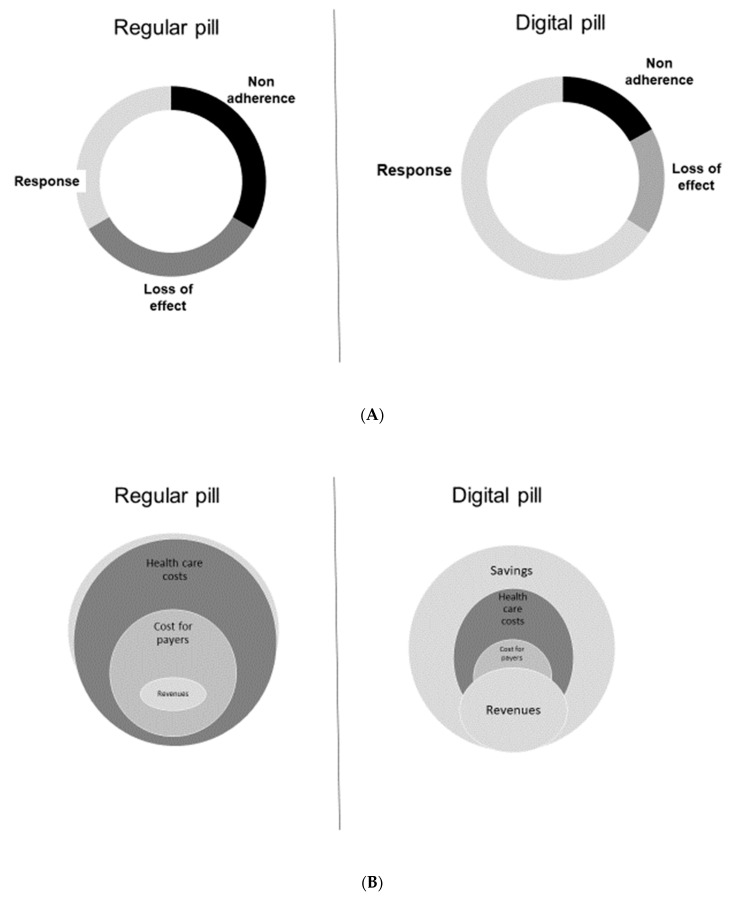

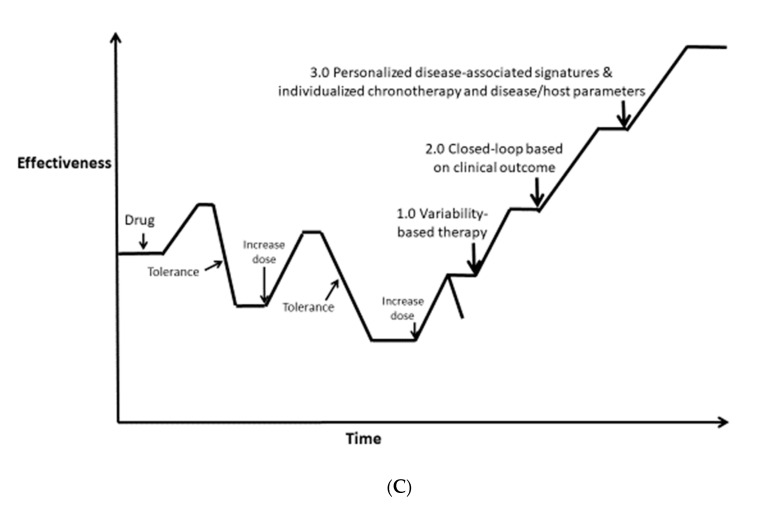
Effect of using the digital pill on overall response rate and market share. (**A**) The use of the digital pill reduces the partial and complete loss of response and improves adherence, increasing the overall response rate to chronic drugs. (**B**) Reducing healthcare costs for the treatment of chronic diseases: Improving the response to chronic medications reduces overall treatment expenses. Savings from using the digital pill can translate into increased revenues for healthcare providers and drug manufacturers, while reducing costs to patients. (**C**) Upscaling the digital pill increases the effectiveness of therapy: Version 1.0, the effect of introducing variability into the therapeutic regimens of patients who stopped responding to chronic drugs was explored using pseudo-random number generators that introduced variability in times of administration and dosages within an approved range. Version 2.0 comprises a closed-loop algorithm that receives inputs based on clinical outcomes. Version 3.0 implements host- and disease-related patterns of variability that are quantified in a personalized manner and implemented into a true-random number generator.

Figure 1 presents a schematic presentation of the advantages of the digital pill, and the beneficial advantages available from savings for users and providers in the healthcare system.

## 8. The Digital Pill as a Market Differentiator for Branded and Generic Drug Companies: Advantages for Patients, Clinicians, Healthcare Authorities, Payers, and Drug Companies

High competition in the overcrowded markets for NCDs is a major challenge for both branded and generic drug companies. Creating a market differentiator for branded drugs usually involves a need to emphasize added value over competitors’ products, which are not always clear. In most cases, for generic companies, price is the main parameter, leading to narrow revenue margins.

The digital pill establishes clear market differentiators for both branded and generic companies. While patient adoption of first generation-based apps has been low, the digital pill’s ability to improve a clinically meaningful outcome is expected to increase adoption by both patients and clinicians. Real-world data comparing the use of fixed dosing regimens with therapeutic regimens can further support the wide implementation of these systems. An improved experience for end-users and healthcare providers using the digital pill is expected to improve sales and enable increased prices.

Contrary to the common scenario of payers and drug companies sitting on opposing sides, the digital pill encourages cooperation. In addition, it creates a market differentiator for drug manufacturers, enabling them to increase market share and product price due to the improved clinical effect of the drug. The increased savings to payers and institutions motivates them to support the use of the digital pill. 

The digital pill is a way to bring all the players in the healthcare system to the same side; patients, clinicians, healthcare authorities, payers, health insurance companies, and drug manufacturers can all benefit from its use. Clinicians and patients will enjoy the clinical benefit, drug manufacturers are expected to increase sales, and healthcare systems will save on costs. This mutually beneficial situation is expected to encourage implementation.

## 9. The Digital Pill as a Basis for a New Intellectual Property

The digital pill, based on the combination of a branded or generic drug with the second-generation AI system, creates new intellectual property (IP) at all stages of development described above. This new IP is a profound market differentiator and can assist companies in increasing their market share and revenues for both branded and generic drugs. 

Each of the stages of development of the second-generation AI system provide a basis for newer IP, enabling companies to increase their revenues as a result of market differentiation. The mutually beneficial model described above is also applicable to the new IP-based digital pill. The advantages of using a digital pill that evolved from an improved response to existing drugs remain applicable when implementing the new IP.

The use of big data by first-generation digital health systems is associated with multiple biases which are commonly introduced into algorithms [42,74,75]. Second-generation AI systems, designed to focus on a single subject, generate data resources that evolve from the effect of treatment on clinically meaningful endpoints. This new type of data resource can serve as a basis for a new IP, as it can support the design of improved algorithms. 

## 10. The Business Model for Implementing the Digital Pill: Win–Win Models for both Users and Providers in Healthcare Systems

The burden of healthcare on the budget of most countries is so significant that it is difficult to receive support from payers and institutions for new entities [76,77]. Introducing a system that improves the response to existing drugs, reduces the economic burden of loss of effectiveness, and reduces the side effects of chronic drugs translates into cost reductions for common NCDs, as well as for rare diseases where current therapies also suffer from a loss of effectiveness. 

The business model for implementing the digital pill has evolved from lowering expenses for medical insurance companies, institutions, and payers. These savings can support the increased revenues of drug manufacturers without increasing the total healthcare budget. Payers can share the cost savings with drug companies, which can benefit from increased product prices and margins. Patients and clinicians share an improved experience without adding costs. Part of the savings can be translated into lower prices for end-users compared with similar competitive medications. These advantages can increase the motivation of all users and providers to use the digital pill.

Contrary to most first-generation AI systems, which introduce new economic burdens on healthcare budgets, second-generation systems are self-sustaining and do not impose additional costs. The costs associated with the development and support of the digital pill are not imposed on the healthcare system. Using the digital pill leads to cost savings for the healthcare system, and a portion of these savings can support the development and maintenance of the second-generation systems. The increase in revenues for drug manufacturers and the savings for insurance companies are expected to support these systems. The digital pill companies share the revenues of the drug manufacturer and/or the savings for the payers. The proposed win–win business model is based on revenues from cost savings for healthcare providers and users alike, and evolves directly from reduced costs.

## 11. Regulation of the Digital Pill 

The regulatory approval for the digital pill parallels the stepwise approach of the different versions of the digital pill. Version 1.0, which is based on open-loop drug reminders, introduces therapeutic regimens that are within the approved therapeutic windows. This system does not collect or generate new data. From the regulatory authorities’ perspective, the use of these systems may be viewed as reminders for improving patient adherence, and may be exempt from regulatory approvals [70]. 

Versions 2.0 and 3.0 use closed loop-based regimens that personalize therapies, and they are required to show superiority over the fixed dosing regimens in controlled trials for their regulatory approval. From the perspective of drug manufacturers, the investment requirement for getting higher versions of the digital pill to market can be supported by the expected increased revenues.

## 12. Conclusions 

Improving global health requires making currently used and newly developed drugs more effective and affordable. The digital pill, which combines a second-generation AI system with a branded or generic drug, improves patient response to chronic drugs by increasing adherence and overcoming the loss of response to these medications. Implementing the use of the digital pill is expected to reduce healthcare costs by assisting both healthcare providers and end-users. Ongoing trials and real-world data using these systems are expected to further support the use of digital pills for improved global health.

## Data Availability

Not applicable.

## Abbreviation

AI	artificial intelligence
